# Paradigm-Specific Risk Conceptions, Patient Safety, and the Regulation of Traditional and Complementary Medicine Practitioners: The Case of Homeopathy in Ontario, Canada

**DOI:** 10.3389/fsoc.2019.00089

**Published:** 2020-01-21

**Authors:** Nadine Ijaz

**Affiliations:** Leslie Dan Faculty of Pharmacy, University of Toronto, Toronto, ON, Canada

**Keywords:** traditional and complementary medicine, epistemology, homeopathy, professional regulation, risk, risk discourse, vitalism, scientific materialism

## Abstract

While the principle of risk reduction increasingly underpins health professional regulatory models across the globe, concepts of risk are neither static nor epistemically neutral. Conventional biomedicine's risk conceptions are substantially rooted in principles of scientific materialism, while many traditional and complementary medicine systems have vitalistic epistemic underpinnings that give rise to distinctive safety considerations. The statutory regulation of traditional and complementary medicine providers has been identified by the World Health Organization as a strategy for enhancing public safety. However, complex risk-related questions arise at the intersection of medical epistemologies whose concepts are at best overlapping, and at worst incommensurable. Elaborating a theoretical concept of “paradigm-specific risk conceptions,” this work employs Bacchi's poststructural mode of policy analysis (“What's the Problem Represented to Be?”) to critically analyze risk discourse in government documents pertaining to the 2015 statutory regulation of homeopathic practitioners in Ontario, Canada. The Ontario government's pre-regulatory risk assessments of the homeopathic occupation discursively emphasized cultural safety principles alongside homeopathy-specific risk conceptions. These paradigm-specific concepts, rooted in homeopathy's epistemic vitalism, extend beyond materialist constructions of adverse events and clinical omission to address potential harms from homeopathic “proving symptoms”, “aggravation,” and “disruption,” all considered implausible from a biomedical standpoint. Although the province's new homeopathy regulator subsequently articulated safety competencies addressing such vitalistic concepts, the tangible risk management strategies ultimately mandated for practitioners exclusively addressed risks consistent with the scientific materialist paradigm. This policy approach substantially echoes the implicit biomedical underpinnings evident in Ontario's broader legislative context, but leaves a significant policy gap regarding the primary safety considerations originally articulated as substantiation for homeopathy's statutory regulation. To optimally preserve patient safety and full informed consent, regulators of traditional and complementary medicine professionals should favor a pragmatic, epistemically-inclusive approach that actively negotiates paradigm-specific risk conceptions from both biomedicine and the occupation under governance.

## Introduction

Significant epistemological challenges arise from the World Health Organization (WHO)'s recommendation that policy makers worldwide implement statutory regulations to govern practitioners of traditional and complementary medicine (T&CM). Traditional medicine systems (such as Chinese medicine and India's Ayurveda), whose indigenous cultural origins pre-date contemporary biomedicine, are rooted in conceptual models that differ significantly from the bioscientific approach. Some complementary medicine approaches (e.g., acupuncture, manual therapies, herbal medicine) originate in such traditional medicine systems, and may retain pre-biomedical epistemic features in contemporary clinical practice. Other, more-recently developed complementary health care systems also have unique epistemic foundations distinct from the conventional biomedical paradigm (e.g., homeopathy), and/or additive upon this paradigm (e.g., osteopathy, anthroposophy, naturopathy) (Ijaz et al., 2019). In an era in which the twin discourses of public safety and evidence-based decision making dominate the global policy sphere (Chamberlain, 2016), how may regulators contend with competing evidentiary claims rooted in health care paradigms whose epistemologies are at best overlapping and at worst incommensurable?

A 2012 WHO survey indicates that regulators seek additional guidance with respect to the implementation of T&CM professional regulations. Among nation states surveyed, 88 and 52% cited “lack of research data” and “lack of [related] expertise” as primary challenges. While an emerging body of literature has begun to document various T&CM professional regulatory processes (see Gale, 2014), few scholars have addressed the epistemic challenges presented in such contexts. Ijaz and Boon (2018b) situate contemporary professional regulation's parameters within a nineteenth century European context, observing that today's dominant regulatory models, “–like biomedicine—bear the epistemic hallmarks of contemporary Western thought.” Calling for regulatory models that address the distinct epistemic features of T&CM practices, they warn:

[T]he predominance of biomedical epistemology and discourse in health professional regulatory structures make regulation of traditional medicine providers a complex prospect rife with potential pitfalls (p. 312).

Elsewhere, with reference to acupuncture's statutory regulation in a North American jurisdiction, Ijaz et al. document one such challenge. Their study shows how policy makers' explicit divorcing of acupuncture from its epistemic roots in Chinese medicine would ultimately promote the misappropriation of traditional knowledge (Ijaz et al., 2016) and unduly privilege biomedically-based acupuncture providers (such as physicians and physiotherapists) in the jurisdiction (Ijaz and Boon, 2018a).

The current work engages with Bacchi's poststructural mode of critical discursive policy analysis (Bacchi and Goodwin, 2016), and the case of homeopathy's recent statutory regulation in Ontario (Canada), to investigate the statutory negotiation of epistemically-pluralistic concepts of risk in regulating traditional and complementary medicine practitioners. The homeopathy case is of particular salience in this context, as its underlying epistemology is widely characterized as implausible from a biomedical standpoint; and may thus be used to highlight the epistemic incongruities potentially at play in T&CM professional regulation. Informed by the theoretical notion of “paradigm-specific risk conceptions,” the study tracks the Ontario government's pre-regulatory risk assessment for homeopathy, the rationale given for homeopathy's regulation, and the safety-related parameters ultimately entrenched in the province's regulatory standards of practice for professional homeopaths. Our analysis shows how state actors may contend with competing evidentiary conceptions within policy frameworks that implicitly privilege biomedical epistemology; and argues that regardless of one's epistemic stance, public safety is best served when paradigm-specific conceptions from a governed T&CM occupation are centered alongside biomedical evidence in policy.

Before turning to the study methods, it is important to provide: (a) an overview of the risk-related theoretical parameters that drive this work; and (b) a brief introduction to the practice of homeopathy.

### Theorizing Risk in T&CM Professional Regulation

Risk may be generally understood as the likelihood of a detrimental outcome occurring (Stub et al., 2015a). A useful theoretical framework for health professional risk assessment differentiates between direct and indirect risk. Direct risk refers to harms of clinical commission, that is, those “caused by medical treatments, procedures and pharmacological products” (Stub et al., 2018, p. 2), and may include: unexpected “adverse reaction[s]” from a “justified treatment”; known “side effect[s]…related to a medicine's pharmacological properties”; and other medical errors (Runciman et al., 2009, p. 22). Indirect risk, by contrast, refers to “a threat to patient safety that is, in the broader sense, associated with the whole treatment setting and clinical practice” (Stub et al., 2018).

One key form of indirect risk is omission – the risk associated with not delivering, or referring a patient to receive necessary care. Prolonged delivery of care that does not appropriately reflect “evidence of effectiveness” may similarly cause indirect harm (Stub et al., 2018, p. 2). Other indirect risks pertain to breaches of professional conduct, including patient consent; and financial, interpersonal or sexual “violation[s]” that arise with deviations from “an operating procedure, standard or rule” (Runciman et al., 2009, p. 23). Culturally unsafe care represents another form of indirect harm, and refers to health care that lacks cultural sensitivity (Anderson et al., 2003) or does not adhere to “patients' values or preferences” (Stub et al., 2018, p. 2).

Notably, the concept of risk is not neutral but culturally- and epistemically-situated see (Ijaz et al., 2016). For example, the potential for harm associated with care that is not culturally safe is a form of risk whose parameters may differ from patient to patient or at the community level, and cannot therefore be conceptualized as absolute or static. Moreover, as elsewhere discussed, risk of harm to patients may be assessed and expressed in narrow, “technical” terms (Ijaz et al., 2016, p. 99) that centralize quantitative measures of tangible events, or more “contextual[ly],” incorporating “a broad range of quantitative and qualitative… sociocultural and ethical factors.” While some risk assessments “mak[e] explicit the[ir] underlying values” (Ijaz et al., 2016, p. 99), this is not always the case. Furthermore, within the context of T&CM professional regulation, divergent conceptions of risk may be epistemically circumscribed by the particular health care paradigms from which they arise.

#### Paradigm-Specific Risk Conceptions

In this work, the term “paradigm-specific risk conception” refers to ways of understanding potential harm that are theoretically endemic to and/or historically expressed within particular health care epistemologies. From this vantage point, concepts of risk that arise from within biomedicine and various T&CM systems are equally paradigm-specific. A key epistemic feature that differentiates biomedicine—and its paradigmatic risk conceptions—from many T&CM epistemologies is its basis in “scientific materialism” (Coulter et al., 2019), a mechanistic “epistemic construct of the human organism” as an “essentially physical entity” (Ijaz et al., 2016). As a result, biomedicine's concepts of clinical efficacy and patient safety emphasize quantitative measures of material events, biological markers, and mechanisms of action. Though not epistemically neutral, scientific materialist conceptions of the human organism are widely taken as axiomatic or universal within our global health systems context of biomedical dominance (Harding, 1998).

Biomedicine's scientific materialism is at paradigmatic odds with “vitalism,” a principle that has been historically central to (although differently articulated within) many T&CM conceptual models including Chinese medicine, Ayurveda, chiropractic, naturopathy, and homeopathy (Coulter et al., 2019). Vitalistic epistemologies generally hold that a “vital” or functional living principle underpins and interacts with the material structures of living systems (including human beings). Vitalistic paradigms have been significantly critiqued over the last centuries with the global ascendance of Descartes' mechanistic worldview (Federspil and Sicolo, 1994), in which “organisms are [understood as] machines and [it is presumed that] everything about them can be explained by the laws of mechanics and physics” (Coulter et al., 2019, p. 61). That being said, a vitalist worldview does not preclude acceptance of material observations about living systems (or about risk of harm *per se*) (Federspil and Sicolo, 1994; Coulter et al., 2019). Rather, vitalist explanatory models may incorporate and contextualize mechanistic observations (about risk and otherwise) with reference to a holistic “operating principle which is not found in inorganic nature” (Coulter et al., 2019, p. 60). Conversely, a scientific materialist stance by definition excludes vitalist perspectives.

Like biomedical concepts of risk, paradigm-specific risk conceptions from within vitalistic T&CM systems may refer equally to direct harms of commission, or to more indirect forms of contextual harm including omission. With reference to Chinese medicine's distinct epistemology and historical canon, Ijaz et al. (2016) have discussed a set of risk concepts intra-paradigmatically associated with the practice of traditional acupuncture. These include but extend beyond tangible physical harm (e.g., lung puncture, bleeding, bruising), addressing such risks as “masking” [or]… “aggravation of symptoms” and “ineffective treatments” (p. 102). The latter risk conceptions are paradigmatically understood to result from treatments that reflect a “poor understanding of Chinese medicine's theoretical principles” (p. 102), such as qi, a vitalistic principle. Paradigm-specific risk conceptions are also evident in homeopathy, and will constitute a primary focus of the present work. Before turning to the homeopathic case, it is useful to introduce a final theoretical concept relevant to the discussion at hand: risk as discourse.

#### Risk as Discourse

Given that all risk conceptions carry implicit or explicit epistemic underpinnings, risk-related evidentiary claims may be better understood as discourse rather than fact. Further, divergent risk discourses may be politically deployed by diverse stakeholders in regulatory contexts, aimed at particular policy outcomes. Wiesener et al. (2018) document stark discrepancies between various European countries' approach to regulating T&CM professionals, ostensibly reflecting differing risk assessments of the same health practices but likely also “based on factors unrelated to patient risk” (p. 6). Elsewhere, Ijaz and Boon (2018a) show how risk discourse may serve as a guise for competing regulatory agendas in a T&CM professional regulatory process, wherein the “malleable” safety principle is unevenly applied to ultimately reinforce biomedicine's epistemic and institutional authority (p. 210–11). It is thus clear that a robust analysis of health professional regulatory risk discourse (such as undertaken in the present work) should concurrently consider matters of epistemology and political context.

### Homeopathic Medicine: A Vitalistic Occupation

Developed by the German physician Samuel Hahnemann (1755–1843), homeopathy is a therapeutic system whose vitalistic epistemic model relies on three primary tenets: (a) the “law of similars”; (b) individualized remedies; and (c) infinitesimal, “potentized” dosing (Owen, 2007). Based on the principle of “like curing like”, homeopathy's law of similars posits that the response produced when a healthy individual ingests a plant, animal or mineral substance provides an indication of the symptoms this substance may therapeutically treat. A homeopathic preparation of onion may for instance be used to treat a person with runny eyes and nose. Homeopathy's individualizing approach furthermore requires that remedies be “matched” to the individual's unique physical, mental and emotional presentation. In other words, the practitioner must select for the best “match” between homeopathic onion and a range of other remedies capable of treating the common cold. Finally, homeopathic medicines, which represent infinitesimal extracts of material substances, are prepared through a systematized process of dilution and shaking. While “low dilution” remedies may contain small quantities of the source materials, “high dilutions” contain no such molecular trace and are considered by homeopaths to exert more potent “energetic” effects.

Several bodies of research interrogate and inform diverse aspects of the homeopathic medical model. Homeopathic pathogenetic trials, or “provings”, have long been used by homeopaths to determine the therapeutic indications for particular homeopathic remedies (Walach et al., 2004; Owen, 2007). Provings involve a “process of administering various highly diluted compounds to healthy consenting volunteers and observing the symptoms induced” (Oberbaum et al., 2012, p. 4). Clinical trials evaluating homeopathy have produced mixed results as to its clinical effectiveness (Linde and Jonas, 1998; McCarney et al., 2004). Many biomedical scientists maintain that homeopathy's proposed mechanism of vitalistic remedial action is physiologically implausible, and represents a therapeutic placebo (Linde and Jonas, 1998). That said, some researchers (e.g., Walach et al., 2008) have interrogated the possibility of non-specific therapeutic effects associated with homeopaths' “in-depth consultations,” which typically extend “beyond the bodily complaints” to include psychological considerations and “lifestyle advice” (Stub et al., 2015b, p. 31–2). Other scholars continue to evaluate multiple theories as to potential mechanisms by which high dilution homeopathic remedies might conceivably exert biological effects (Khuda-Bukhsh, 2014).

Across the globe, homeopathy is one of the most widely-practiced T&CM approaches and is variously integrated into state health systems. Where related statutory regulation exists, it generally involves policies governing homeopathy as practiced by licensed physicians and/or non-physician practitioners (see Table 1). The current study presents an analysis of risk discourse with reference to the recent statutory regulation of homeopathy in the Canadian province of Ontario, Canada. North American homeopaths were widely regulated in the nineteenth century (O'Reilly, 1999), and licensed naturopathic physicians across much of the US and Canada today include homeopathy in their scope (American Association of Naturopathic Medicine, 2018). In 2013, however, Ontario became the first jurisdiction on the continent to regulate non-physician homeopaths in the present day (Ijaz et al., 2015).

**Table 1:** Homeopathic practitioner regulations across the globe.

**Laws governing homeopathic practice by licensed physicians**	**Laws governing non-physician homeopathic practitioners**
Argentina, Austria, Belgium, Brazil, Bulgaria, Czech Republic, Colombia, Costa Rica, Croatia, Denmark, Ecuador, Estonia, France, Germany, Greece, Hungary, Iran, Italy, Latvia, Liechtenstein, Lithuania, Mexico, Romania, Slovenia, Spain, Switzerland, Turkey, USA (some states)	Canada (Ontario only), Croatia, Denmark, Estonia, Germany, Greece, India, Pakistan, Poland, Portugal, South Africa, Sri Lanka, Turkey

*Wiesener et al. (2012)*.

## Methods

The present study evaluates the epistemic construction of risk in policy-related texts pertaining to homeopathy's statutory regulation in the Canadian province of Ontario, using Bacchi's “WPR” approach to critical policy analysis (Bacchi and Goodwin, 2016). The WPR method critically interrogates the construction of public policy issues by asking “What's the Problem Represented to Be”? As a poststructural analytic method informed by the work of Foucault, WPR fundamentally contests the “common assumption in many approaches to policy analysis that ‘problems’ simply exist and that their meaning is clear and uncontentious” (p. 12). Rather, as Bacchi explains:

The “WPR” approach …starts from the premise that what one proposes to do about something reveals what one thinks is problematic (needs to change). Following this thinking, policies and policy proposals contain implicit representations of what is considered to be the “problem” (“problem representations”) (Bacchi, 2012, p. 21).

WPR takes as axiomatic that no evidentiary claim made in support of public policy is neutral. As such, the method—which proceeds in six steps—emphasizes analysis of the epistemic and/or political underpinnings of particular policy proposals, claims or structures, to critically expose the “unexamined assumptions and deep-seated conceptual logics within implicit problem representations” (p. 22). Bacchi's six steps address (though not necessarily in sequence): (1) characterization of the constructed policy problem; (2) identification of the problem's conceptual underpinnings; (3) evaluation of its origins; (4) problematization of the problem, including its silent or implicit features; (5) consideration of the problem construct's implications; and finally, (6) discussion of public consequences of the policy problem as-constructed, and identification of policy alternatives.

The present study engages with the WPR method to critically interpret the central construction of “risk” as a primary focus in two distinct bodies of policy-related text. The first set of “pre-regulatory” textual excerpts is drawn from a single 2006 government report by Ontario's Health Professions Regulatory Advisory Council which, in response to a request for study by the province's Health Minister, recommended regulation of Ontario's homeopathic practitioners. In the second set of texts are policy documents published by Ontario's new homeopathy regulator in relation to the implementation of such regulations in 2015. These include two documents outlining competency and performance indicators for regulated homeopaths, as well as 19 distinct practice standards and six professional conduct guidelines outlining the regulator's expectations of registered homeopaths. Across these texts, the study applies the theoretical lens of “paradigm-specific risk conceptions” aims to: (a) unpack the risk-related rationale initially provided for homeopathy's regulation in Ontario, and (b) the safety-related parameters ultimately entrenched in the province's homeopathic regulatory framework.

To set the stage for this analysis, an overview follows of paradigm-specific risk-related considerations pertaining to homeopathy.

## Risk and the Practice of Homeopathic Medicine

**Figure 1:**
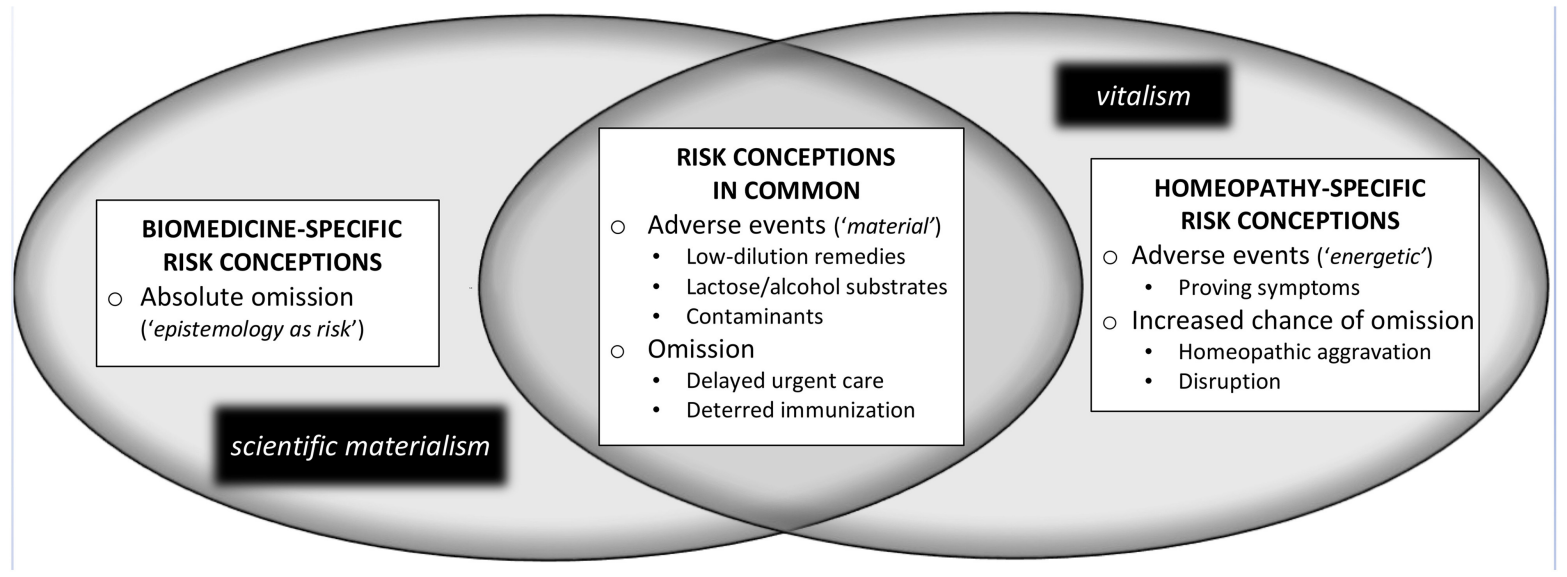
Risk conceptions associated with homeopathic clinical practice.

The example of homeopathy clearly demonstrates how the unique risks associated with clinical practice in a particular T&CM occupation may be differentially conceptualized. As shown in Figure 1, distinct risk conceptions associated with homeopathic clinical practice are evident from both a biomedical and homeopathic epistemic standpoint, although–owing to vitalism's inclusion of material considerations–there are also shared concepts of risk across these distinct positionalities. These distinct risk conceptions are presented below with reference to related scholarly and historical literatures by drawing attention to their epistemic underpinnings and potential policy implications. It should be noted that the following discussion focuses on risks specifically associated with homeopathic clinical care, in this case pertaining to the direct risk of adverse events, and the indirect risk of omission. This account does not address matters of professional conduct (e.g., financial or sexual abuse) that are more generally relevant across health occupations. The first homeopathy-related risk discourse discussed in what follows is biomedically-underpinned, prominent across the scholarly and gray literature, and critiques homeopathy in absolutist terms.

### Absolute Omission: A Biomedical Discourse of Epistemology-as-Risk

As shown to the far left of Figure 1, a particular discourse –which I term “absolute omission”– is frequently evident in biomedicalized accounts of homeopathy's associated risks. This discourse holds that “homeopathy is unethical on the basis that its knowledge claims are not commensurable with scientific principles, particularly those of evidence-based medicine” (Levy et al., 2015). As Ernst, a scholar, biomedical doctor and public critic of homeopathy, argues (Fisher and Ernst, 2015, p. 2):

As the typical homeopathic remedy is devoid of active molecules, it is unlikely to cause serious adverse effects. However, even a placebo can cause harm, if it replaces an effective [biomedical] therapy. …Even [homeopathy's] use as a “benign placebo” for self-limiting conditions is problematic. In such cases it would be preferable to reassure patients rather than to deceive them with placebos.

This discourse, which takes homeopathic epistemology as a source of risk and constitutes biomedical knowledge as universal, holds that any homeopathic care is harmful homeopathic care since it displaces “authentic” biomedical care. In other words, the act of providing homeopathic treatment is constructed in absolutist terms as an act of omission. As (Ernst, 2019, p. 1) asserts, “all clinicians who prescribe homeopathy are guilty of neglect. …the damage done is merely a question of degree.” Further from this vantage point, any potential benefit that a patient may derive from homeopathic care is viewed as resulting from the placebo effect, framed in this context as an unethical form of harm-by-deception.

The a priori construction of homeopathic care as professional misconduct has a regulatory corollary, as exemplified in an editorial in the British Medical Journal. Addressing the prospect of regulating T&CM providers in the United Kingdom, Colquhoun—a biomedical researcher—characterizes homeopathic remedies like Ernst as “medicines that contain no medicine whatsoever” (Colquhoun, 2009, p. 2). He consequently argues that homeopathy be absolutely precluded from professional regulatory consideration (p. 2):

You cannot start to think about a sensible form of regulation unless you first decide whether or not the thing you are trying to regulate is nonsense.…[T]he statutory regulation of things that don't work endangers patients.

The biomedicalized discourse of absolute omission presented above has been critiqued by ethicists as “exaggerated, unsupported and illogical” on the basis that its claims are “founded on unsophisticated notions of evidence, that adopt narrow perspectives on health care assessment, and that overstate the personal, social and ontological harms of homeopathy” (Levy et al., 2015, p. 207–8). This discourse is also at odds with a growing body of literature that points to potentially-advantageous aspects of the so-called placebo effect (Kaptchuk, 2018). Also significant is how this discourse exemplifies biomedicine's universalizing epistemic “monotheism,” which as Hollenberg and Muzzin (2010, p. 52) note, “prevents… tolera[nce of] alternative paradigms.” Regardless, a discourse of homeopathic absolute omission remains influential in the scholarly, clinical and public spheres. That being said, even from a biomedical standpoint it becomes possible to take a more epistemically “agnostic” or “inclusive” approach to evaluating homeopathy's associated risks and benefits if one recognizes that from an epistemological point of view both vitalism and mechanism [scientific materialism] are metaphysical doctrines and that neither can be submitted to experimental control (Federspil and Sicolo, 1994, p. 342).

### Homeopathic Risks in Common: Epistemic Overlap

Toward the center of Figure 1, a set of homeopathy-related risk conceptions is presented that are rooted in “material” considerations, but generally taken as relevant from both biomedical and homeopathic epistemic standpoints. These risk conceptions, as discussed in the scientific literature, address the potential for both direct and indirect harms associated with homeopathic care.

With respect to the direct adverse events from homeopathic remedies, it is generally agreed upon biomedically (Posadzki et al., 2012) and homeopathically (Stub et al., 2016) that low-dilution homeopathic remedies may cause allergic or toxicological (i.e., “material”) adverse responses “if administered too frequently over a long period of time” (Stub et al., 2015b). This risk relates to the presence of trace quantities of the original medicinal substances in low dilution homeopathic remedies, and is of particular concern when the original substances have toxic effects (Posadzki et al., 2012). To address such direct risks, several jurisdictions, including the European Union, Canada and Australia, have implemented regulations that specify “safe” dilutions for commercial homeopathic preparations (Buchholzer et al., 2014). Also epistemically-uncontentious is that homeopathic medicine users may react adversely to a remedy's substrate, typically a pill composed of lactose (Endrizzi et al., 2005), or a solution of alcohol (Oberbaum et al., 2012). Another material risk discussed in the literature is that of remedy contamination (Buchholzer et al., 2014), which may occur either in commercial contexts or when homeopaths compound individualized remedies from raw substances. The WHO has published an international guideline on the manufacture of safe homeopathic products, to address the aforementioned issues.

Further consistent with both a homeopathic and biomedical standpoint is the potential for harms of omission. In contrast to an exclusively-biomedical discourse of absolute omission, this shared conception of omission has two primary subcomponents. The first pertains to delays of urgent or otherwise necessary care when a homeopath fails to appropriately refer a patient to another health care provider; individual cases of this form of omission have been documented in the literature (Posadzki et al., 2012). The second type of omission scenario evident in this area of epistemic overlap refers to the potential for patients to defer or decline immunizations as a result of treatment with a type of homeopathic remedy referred to as “nosodes.”

Nosodes are homeopathic dilutions of inactivated disease agents, which some homeopathic practitioners recommend to patients with the vaccine-like aim of thereby preventing infectious disease (“homeoprophylaxis”) (Roniger and Jacobs, 2010). Although there is a long history of nosode usage in homeopathy (including non-prophylactic applications), bioscientific evidence for homeoprophylaxis is limited (Jonas, 1999). From a biomedical perspective, homeoprophylaxis has been characterized as representing a significant public health risk:

There is no good evidence that any form of homeoprophylaxis is effective.… The promotion of this approach constitutes a serious risk for public health: once rates for conventional immunisations fall below a certain threshold, the population would lose its herd immunity (Ernst, 2016, p. 1).

It should be noted that homeoprophylaxis is not the norm in contemporary homeopathic practice (Burgess, 1994; Roniger and Jacobs, 2010), and that many homeopathic practitioners, researchers and advocates decry the practice for similar reasons to those outlined by Ernst above (Fisher, 1990; Burgess, 1994). Omission related to homeoprophylaxis may thus be understood as an area of partial agreement between some homeopathic proponents and their biomedical counterparts.

### Homeopathy-Specific Risk Conceptions: Proving Symptoms, Aggravation, and Disruption

As now discussed, however, there are three primary concepts of homeopathic risk discussed in the literature that—owing to their vitalistic underpinnings–are best described as homeopathy-specific from an epistemic standpoint. These three concepts—proving symptoms, homeopathic aggravation and disruption – give rise to the potential for specific direct harms (i.e., adverse effects) as well as indirect harms (i.e., a particular form of omission) to occur within the context of homeopathic care. In contrast to the material forms of harm discussed to this point, the three aforementioned concepts pertain to the “energetic” action understood by homeopaths to be exerted on the patient's vital force in response to particular homeopathic remedies. To better contextualize these risks, I refer to Stub et al.' paradigm-specific definition of adverse events in homeopathy as “undesirable effects of a remedy” (Stub et al., 2012b, p. 17). This definition permits differentiation of clinical outcomes that might be “unexpected and/or unpleasant but welcome” from those that are considered “undesirable” and thus “adverse.”

#### Proving Symptoms

Proving symptoms draw their name from homeopathic pathogenetic trials, or “provings,” in which healthy volunteers consent to take low- or high-dilution homeopathic remedies and document the symptoms that arise as a result (Walach et al., 2004; Owen, 2007). Proving symptoms may be experienced as mild, moderate or severe in intensity; and are typically understood to abate once the volunteer ceases repetitive dosing. Such symptoms are considered “desirable” in the context of a proving, which aims to investigate the therapeutic indications for a particular homeopathic medication. In a clinical care context however, similar proving symptoms—which do not specifically ameliorate the patient's health condition—may also arise. Here, according to Stub et al.'s definition (2012b), such proving symptoms would be conceptualized as “undesirable” adverse events (From a biomedical standpoint, these same symptoms would be constituted as placebo or nocebo responses, unless materially-associated with a low-dilution remedy).

The evidentiary evaluation of adverse events from homeopathic proving symptoms problematizes what researchers may consider credible epistemic constructs around risk. Some clinical studies have explicitly documented incidence of mild or moderate proving symptoms in clinical settings (e.g., Endrizzi et al., 2005; Michalsen et al., 2015), a finding that takes non-material effects as epistemically credible. A 2016 systematic review and meta-analysis of randomized controlled trials of homeopathy (*n* = 39) showed mild and moderate adverse events to occur equally from low and high dilution remedies (Stub et al., 2016). This review also showed homeopathic adverse events to occur at a rate similar to those adverse events in placebo-controlled trials of conventional medicine, lending credibility to its epistemically-pluralistic findings.

However, a prior systematic review adopted a strictly biomedical epistemic posture and concluded that “plausible” homeopathic adverse events were those reported from the “material” effects of low dilution remedies alone (Posadzki et al., 2012, p. 1186). That being said, another recent study concluded –after extensive but negative toxicological analyses– that “a high frequency of apparent life-threatening events” among “infants treated with [a] complex homeopathic medication” (Oberbaum et al., 2012, p. 3) appeared unexpectedly consistent with “the purported action of proving as described in homeopathy” (p. 8). On this basis, the study authors recommended additional laws to govern “qualification of those practicing homeopath[y]” (p. 9).

#### Homeopathic Aggravation

Sometimes termed a “healing response,” homeopathic aggravation refers to a short-term, mild or moderate worsening of a patient's health condition that provides a positive indication that the therapeutic process is underway (Stub et al., 2012a). Hahnemann, homeopathy's founder, explains this paradigm-specific concept in the Organon of Medicine, one of the profession's core texts:

This aggravation is so similar to the original disease that to the patient it appears to be an aggravation of his [sic] own complaint … This slight homeopathic aggravation in the first hours is a good portent…and is not unusual (Hahnemann et al., 1982, §157-8).

Clinical research assessing the frequency of homeopathic aggravation has yielded varying results, with reports ranging from 10 to 75% of cases (Stub et al., 2015b). In one qualitative study, most homeopaths “claimed to have observed aggravations in 60–70% of their cases” (Stub et al., 2012b, p. 15); and, in a survey of homeopathic patients, 26% reported minor (66%) or moderate (34%) worsening of symptoms after receiving homeopathic treatment (Stub et al., 2015a). Given that aggravation is conceptualized in homeopathy as a “desirable” response to treatment, it would not necessarily be viewed as an adverse event. Biomedically, a similar turn of events might be viewed as unrelated to treatment, or as a nocebo response if patients had previously been advised that aggravation were a possibility (Stub et al., 2015b).

The primary risk associated with homeopathic aggravation is that of omission, in which a provider might inappropriately interpret prolonged or severe symptoms as positive treatment indicators, thus delaying or failing to refer the patient to other necessary care. To manage this risk, Stub et al. (2012a) propose a time-limited definition for homeopathic aggravation with two key stipulations: (a) worsened symptoms that follow the initial remedy must be accompanied by an improved sense of well-being within 3 days; and (b) the aggravation itself should last no longer than 14 days. Otherwise, they propose, aggravated symptomatology should be interpreted as an adverse event and/or trigger referral. That said, such a definition is at somewhat at odds with traditional homeopathic theory, in which an aggravation may reasonably extend over several weeks or months (Stub et al., 2015a).

#### Disruption

Disruption, finally, is another homeopathic theoretical concept that refers to a reaction following an incorrect remedy…[that] causes disappearance of some symptoms and the creation of new symptoms, and is frequently seen in clinical practice…[when the patient] is *sensitive* [emphasis added] to the medication (Stub et al., 2012a, p. 5).

The concept of sensitivity in homeopathic epistemology, mentioned in the cited excerpt, refers to a situation in which a remedy is “well-matched” to a particular individual, and thus provokes a therapeutic response in the organism. In disruption, a remedy is viewed as sufficiently well-matched to provoke a partial therapeutic response, but not adequately matched to fully resolve the symptom picture. As Hahnemann, homeopathy's founder, explains, the new symptoms that emerge in disruption usefully provide the clinician with indicators in selecting a more appropriate second remedy:

[I]f there occur, during the use of this imperfectly homeopathic remedy first employed, accessory symptoms of some moment, then…we investigate afresh the morbid state in its now altered condition, and add the remainder of the original symptoms to those newly developed in tracing a new picture of the disease. (Hahnemann and Trans. Boericke, 1921, §167).

Understood thus as a desirable response along the therapeutic trajectory, disruptions would not be seen as adverse events by homeopathic practitioners (Biomedically, the “new symptoms” of disruption might be “defined as adverse events”, Stub et al., 2012a, p. 7) if associated with a low-dilution remedy; or alternately, as entirely unrelated to the homeopathic intervention).

Although poorly-documented in clinical studies, disruption appears to be a phenomenon well-recognized by contemporary homeopathic practitioners (Stub et al., 2012a); it thus warrants consideration in homeopathic risk assessment. Distinct from homeopathic aggravation, disruption is not typically accompanied by the onset of feelings of well-being, headache and/or insomnia in addition to the new presenting symptoms. As with aggravation, the primary risk associated with disruption is omission, should its concomitant symptoms become severe or prolonged.

## Results

### The Context of Homeopathy's Regulation in Ontario

In 2006, the Ontario government announced its decision to regulate homeopathy under the province's Regulated Health Professions Act (RHPA), and the province's homeopaths would ultimately be regulated in 2015. Using a statutory self-regulatory model that governs 26 Ontario health professions, the RHPA authorizes restricted titles to specific health occupations while permitting overlapping scopes between them (HPRAC, 2006). In this model, some professions are furthermore granted access to particular “controlled acts” that are otherwise restricted from the public domain (O'Reilly, 1999). While the RHPA is an epistemically-inclusive piece of legislation that governs biomedically-trained providers as well as T&CM professionals (e.g., Chinese medicine practitioners, naturopaths) the RHPA's handling of one of its controlled acts—“communication of a diagnosis”—makes clear that the legislation is not epistemically neutral. While this controlled act is authorized to several professions with varying restrictions, it is only with reference to Ontario's Chinese medicine professionals that an epistemic qualifier is added to the controlled act, to read “Communicating a traditional Chinese medicine diagnosis” (Government of Ontario, 2015a,b, § 4-2). It is thus clear that biomedical diagnosis (and with it, biomedical epistemology) are constituted as normative under the RHPA.

In early 2005, following requests from Ontario's homeopathic practitioner community, the province's Health Minister called on the Health Professions Regulatory Advisory Council (HPRAC) to evaluate whether “homeopaths should be regulated” (HPRAC, 2006, p. 294). As an arms-length governmental body that provides health professional regulatory advice, HPRAC conducts studies to prepare reports that respond to the Minister's requests. Central to HPRAC's mandate, aligned with global “risk-based regulation” trends (Lloyd-Bostock and Hutter, 2008), is the principle of public protection (HPRAC, 2006). HPRAC undertook to prepare such a report on homeopathy informed by a literature review, a dozen “key informant” interviews, and “97 [written] submissions” from various stakeholders (p. 157). Delivered to the Minister in 2006, and predicated on safety-based grounds, HPRAC's report recommended statutory regulation of Ontario's homeopaths. HPRAC's report on homeopathy was embedded in a larger document entitled New Directions, which responded to a series of distinct requests made by the Minister in his letter of February 2005.

HPRAC's approach to developing recommendations would likely have been informed by a number of political factors, which the current study will not interrogate at length. For example, in a second letter to HPRAC also appended to the New Directions report, Ontario's health minister notes: “Our government is committed to ensuring that users of non-traditional medicine and alternative approaches will have confidence in their safety” (p. 299). Although this second letter addresses issues related to the forthcoming regulation of Ontario's Chinese medicine practitioners rather than homeopaths specifically, it exposes a political agenda on the provincial government's part that favored T&CM professional regulation at the time. HPRAC's awareness of such an agenda is elsewhere evident in the New Directions report, which—in addition to its homeopathy-related recommendation—proposes that another Ontario T&CM profession, naturopaths, also be regulated under the RHPA. HPRAC had twice before studied the naturopathic question (in 1996 and 2001, respectively), recommending regulation on each occasion; however, no related legislative action had followed. Notably, the Minister did not directly request naturopathy-related advice in his 2005 letter to HPRAC. However, Ontario's naturopathic occupational leaders are explicitly “carbon-copied” on this ministerial letter, demonstrating a political imperative—which HPRAC clearly recognized–to advance naturopathy's professional regulatory project in the province.

The aforementioned points affirm that the texts under analysis represent strategic political discourse, which the interpretation in what follows takes as axiomatic but does not further interrogate. Rather, the present work investigates the ways in which divergent or overlapping conceptions of risk from within distinct medical epistemologies may be expressed as political discourse. Part I of the Results segment shows how HPRAC's 2006 report would strategically elevate homeopathy-specific risk conceptions and submerge biomedical critiques of homeopathy's vitalistic epistemology to construct a viable justification for homeopaths' future statutory regulation under the RHPA. Part II demonstrates how the risk management policies ultimately implemented as part of Ontario's homeopathic practitioner regulations would subordinate vitalistic risk conceptions to matters exclusively material, considerably at odds with HPRAC's recommendations and the RHPA's epistemically-inclusive character, but closely aligned with other related provincial legislation.

### Part I: The HPRAC Report

#### HPRAC's Culturally-Situated, Epistemically-Inclusive Approach

In the preamble to its New Directions report, HPRAC constructs the delivery of culturally-safe care as a key regulatory consideration. Situating T&CM care within the broader context of “demographic change in Ontario” HPRAC (2006, p. 10), cites immigrant influxes:

For many [newcomer Ontarians], the use of safe complementary or alternate care is part of their experience, cultural heritage and way of life, and a preferable method of treatment over conventional medicine. They do, however, expect that practitioners who are providing their care are qualified and meet the standards of practice of the alternate form of medicine (HPRAC, 2006, p.12).

One-third of the homeopathic practitioners eventually regulated under the RHPA's Homeopathy Act (and we suggest, a high number of Ontario's homeopathy users) would be immigrants from South Asian countries such as India and Pakistan (Ontario Fairness Commissioner, 2018), where homeopathy has long been regulated. HPRAC's implicit centralization of this point signals that its risk assessment for homeopathy will address contextual, rather than exclusively technical evidentiary matters that take into account diverse cultural worldviews.

Also in the report's preamble, HPRAC directly addresses the epistemic and evidentiary “challenges” raised by its study of homeopathy. Central to its study, HPRAC notes, was “[t]he question of whether a profession needed to prove the efficacy of its treatments” to be eligible for statutory self-regulation in the province (p. 92). In this regard, HPRAC explicitly enumerates significant features that differentiate homeopathy from conventional biomedicine, such as its reliance on “‘provings’ that are based on a holistic approach to health care and the ‘law of similars’ as observed in individual patients” (p. 92). Centering the safety principle, HPRAC ultimately concludes that a health occupation's particular worldview or concomitant therapies should not constitute professional regulatory criteria under the RHPA:

[T]he RHPA does not regulate a therapy or a therapeutic approach. It does, however, regulate individuals who practice a form of health care – whether conventional, complementary or alternative – and provides a safeguard for patients or clients. (p. 92–93).

HPRAC's epistemically-inclusive approach would however remain at odds with the views expressed by some of its consulted stakeholders.

#### Subdued Biomedical Voices, Amplified Homeopathic Perspectives

As part of its study, HPRAC heard from a wide range of stakeholders including “educational institutions, associations, regulatory bodies and individuals” (p. 157), among whom were biomedical critics of homeopathy. Early on in the New Directions report, HPRAC observes that

[H]omeopathic principles are not accepted by all. A significant number of conventional medical practitioners, allied professions and clinical scientists seriously question the efficacy of homeopathy and regard it as unsafe. They point to the fact that there is no body of evidence that shows that homeopathic principles when translated into practice are efficacious. (p. 152).

This passage exemplifies a risk discourse of “absolute omission,” in which homeopathic epistemology is itself constructed as a source of risk to patients. As noted earlier, and at odds with the Health Minister's directive, such a position precludes – on principle– consideration of homeopathy's suitability for statutory regulation. By giving voice to this epistemically-biomedical stance early on, HPRAC effectively recognizes the voices of stakeholders holding such a view. However, across the remainder of the report's 22 page segment on homeopathy, HPRAC holds to its epistemically-inclusive approach and alludes no further to homeopathy's purported incommensurability with biomedical science. Although HPRAC's homeopathic report is otherwise replete with citations to textual sources, no such references accompany the above-cited passage. It may thus be inferred that the views represented in that passage were those of biomedical stakeholders consulted as part of HPRAC's study, whose voices HPRAC ultimately subdues. Across the report's pages, conversely, HPRAC amplifies the range of perspectives expressed by homeopathic stakeholders, including homeopathy-specific perspectives on risk.

While “various factions” (p. 157) within Ontario's homeopathic community are reported to have held a number of internally-conflicting views, HPRAC characterizes homeopathic stakeholders as substantially agreeing in their characterization of their occupation's associated risks. In HPRAC's account, these stakeholders delineate homeopathic remedies as “generally safe.” That being said, they recognize a “largely anecdotal” potential for material “direct harm” to occur in relation to the “improper [hand-made] dilutions of ‘mother tinctures’ of homeopathic remedies by unqualified practitioners” (p. 158). Demarcating “indirect harm” instead as their occupation's primary safety issue, homeopathic stakeholders reportedly express “general agreement” that more “serious risks to the public” could arise from the “misappl[ication of] homeopathic principles” as well as “incorrect assessment, failure to refer or fraud” (p. 158).

Aside from fraud (associated with professional misconduct), this particular risk discourse is substantially consistent with homeopathy-specific concepts. Indeed, both the notions of “misapplication” and “incorrect assessment” represent homeopathy-specific ideas about the outcomes–such as proving symptoms and disruption–that may arise when homeopaths prescribe remedies poorly “matched”–another homeopathic concept–to patients. That “failure to refer” (in other words, omission) is cited alongside these homeopathy-specific principles suggests inclusion of yet another epistemically-situated concept—homeopathic aggravation—in stakeholder accounts, as HPRAC subsequently affirms in its own synthetic risk assessment.

#### HPRAC's Epistemically-Inclusive Risk Synthesis

Addressing the Minister's question as the report advances, HPRAC argues that “there is a risk of both direct and indirect harm” to the public associated with homeopathic practice; and that “[i]n the absence of regulation,” such risks are unnecessarily “heightened” (p. 163). HPRAC substantiates this risk-based regulatory recommendation by again emphasizing homeopathy-specific epistemic concepts (both material and energetic) over exclusively biomedical perspectives. As in the broader report, HPRAC's risk synthesis begins with a cursory nod to biomedical stakeholders, briefly addressing a discourse of epistemology-as-harm in which “the homeopathic approach” is seen to present a potential risk to the public. In a single sentence, HPRAC summarizes the key harms associated with such a stance as “prevent[ion] of other effective (medical) interventions” and “discouraging immunization.” The remainder of HPRAC's two-paged risk synthesis segment, however, presents a narrative that cites peer-reviewed and gray literatures to substantially echo homeopathic stakeholders' own risk discourse.

While HPRAC does not provide an explicit definition of “adverse reactions” in the context of homeopathic practice, it includes as “direct risks” both material and energetic safety considerations (p. 163). Cited material risks include “allergic reactions to low potency homeopathic preparations” and dispensing of “potentially toxic” dilutions of “arsenic and cadmium”; remedy “misapplication,” an energetic concept (as discussed above) is also included as a potential direct harm (p. 163). While HPRAC's enumeration of possible “[i]ndirect risks” lists such biomedically-consistent concepts as “missed diagnoses” and “delay of effective therapy,” the list also includes such homeopathy-specific energetic concepts as “interference of remedies with conventional treatments,” a notion that recognizes the (biomedically-inconceivable) potential for homeopathic remedies to interact with pharmaceutical medications (p. 164).

Further illustrating HPRAC's attention to energetic considerations, the text provides considerable detail about the potential for “‘prolonged suffering…from homeopathic “aggravations” or “healing crises” where symptoms become worse before improving”’ (p. 164). HPRAC specifically cites peer-reviewed studies that document aggravation (“return of old symptoms”) as well as disruption or proving symptoms (“new symptoms”) over the course of homeopathic care; and draws attention to one such study's conclusion that recording side effects would facilitate broader understanding and enable standards to be set for information audits and patient care (p. 164). The message thus conveyed to the Minister and future policy makers is that: (a) homeopathic care is sufficiently risky to regulate; and (b) future regulations should explicitly address both material and energetic risks, whether direct or indirect, associated with homeopathic practice.

### Part II: Risk and Ontario's New Homeopathy Regulations

In 2015, almost 10 years after Ontario's Health Minister commissioned HPRAC to study the issue of homeopathy's professional regulation, the province's Homeopathy Act (2007) came into force under the RHPA (Government of Ontario, 2007). No controlled acts were included in the new homeopathic profession's practice scope, signaling that the profession's range of clinical approaches was on the whole relatively safe. Under the auspices of a new regulatory body—the College of Homeopaths of Ontario (CHO)—Ontario's homeopaths would now be required to practice in compliance with a detailed set of competency and performance indicators, adherence strategies for which are detailed across 19 practice standards and six professional conduct guidelines.

Among the CHO's performance indicators for homeopaths are thirty-six “safety competencies” identified as “those most important to minimizing the risk of harm to the patient” (Transitional Council of the College of Homeopaths of Ontario, 2012c). As shown in Table 2, about one-third of these safety competencies hold general relevance for all health professionals, whereas the remaining 22 refer to homeopathy-specific occupational skills constituted by CHO to carry a risk of harm. Using a theoretical lens that centralizes paradigm-specific risk conceptions in homeopathy, the analysis below evaluates these safety competencies and the CHO standards and guidelines that address them.

**Table 2:** Safety competencies for regulated homeopaths in Ontario, Canada.

**Competency type**	**Theme**	**Inclusions (and competency citation)**
General health professional competencies (*n* = 14)	Professional conduct (*n* = 6)	Scope adherence (1.1); Working within individual skillset (1.4); Privacy and confidentiality (1.5); Informed consent (2.25); and Maintenance of physically-safe clinical environment (2.46, 3.1)
	Clinical skills (*n* = 5)	Attending to patient values/preferences (1.3, 1.6); Physical examination skills (2.20, 2.28d); and First aid skills (2.24)
	Interprofessional collaboration/omission (*n* = 3)	Seek appropriate professional advice to compensate for knowledge gaps (1.10); Recognize and refer for serious/life-threatening conditions (2.17); and Refer/collaborate to provide optimal patient care (2.46)
Competencies unique to homeopathic practice (*n* = 22)	Clinical skills (*n* = 22)	Homeopathic patient intake process (2.28a, c; 2.19); Individualized differential diagnosis (2.1b, d, f; 2.4); Remedy selection, administration and dispensing (2.1a, c; 2.23; 2.37; 2.7a, b); Documentation of patient cases (2.41) and patient instructions (2.39 a, b, c, d, e); Follow-up case management (2.42, 2.43)

*Transitional Council of the College of Homeopaths of Ontario (2012a,c)*.

As discussed earlier (and shown in Figure 1), there are two primary types of risk associated with homeopathic practice—adverse events and omission; and, crucially, each of these risk types may be differentially understood from a biomedical and/or homeopathic epistemic standpoint. It is clear that the CHO did not adopt into policy the biomedical concept of absolute omission, which would have precluded implementation of regulations governing professional homeopaths. Setting aside matters of basic professional conduct relevant for all health professionals, the following analysis shows how the CHO's policy documents engages in turn with biomedical and homeopathic risk conceptions regarding adverse effects and (non-absolute) omission across its safety competencies. In the standards and guidelines that mandate how homeopaths deploy these competencies in practice, however, the CHO implicitly renders homeopathy-specific risk conceptions inconsequential, while reinforcing the institutional dominance of biomedicine's scientific materialism as elsewhere entrenched in Ontario law. The latter point becomes most apparent in the CHO's policy on informed consent, with which the analysis below begins.

#### Adverse Events as Material Events

Among the performance indicators stipulated for the CHO's safety competencies is a requirement that homeopaths advise their patients, “in writing, [of] the cautions and warnings associated with taking the [homeopathic] medicine” (Transitional Council of the College of Homeopaths of Ontario, 2012c, p. 32). This requirement is further affirmed in the CHO's practice standards, which mandate that homeopaths “obtain informed consent prior to proceeding with a treatment plan” (College of Homeopaths of Ontario, 2018a, p. 1). Details about how this requirement may be met are provided in a stand-alone standard on informed consent, wherein homeopaths are instructed to ensure that

the patient understands and appreciates the nature, anticipated benefits, material risks and side effects [emphasis added] … of the proposed intervention and agrees to proceed it” (College of Homeopaths of Ontario, 2013a, p. 4).

The italicized portions of this passage are significant in that they entrench into policy an exclusively biomedical perspective on risk, in which scientific materialist conceptions of homeopathic remedies (and their potential associated harms) supersede the vitalistic risk conceptions characteristic of homeopathic epistemology. In other words, this passage implicitly gives unique importance to such “material risks” as allergic or toxicological responses, while concurrently discounting possible “energetic” adverse events (e.g., proving symptoms). Indeed, if the CHO had wished to convey that such “energetic” risks were “real,” it is likely they would have required that patients be informed thereof. Although the CHO nowhere provides an explicit definition of “adverse event” in its policy documents, the wording used in its informed consent standard may understood to implicitly represent such a definition.

Importantly, the “material risks” wording (and, ultimately its biomedical underpinnings) reflect phrasing elsewhere legally codified in Ontario's Health Care Consent Act. Below, on the left and right, respectively, we show the notable similarities between the CHO's sample informed consent form and the aforementioned provincial legislation are shown (with italics added for emphasis):

**Table:** 

…I have been informed of, and understand…	A consent to treatment is informed if, before giving it, the person received the information about the [following] matters [:]
the nature of the procedure,	The nature of the treatment.
expected benefits,	The expected benefits of the treatment.
*material risks*,	*The material risks of the treatment*.
*material side effects* and financial cost…	*The material side effects of the treatment…*
(College of Homeopaths of Ontario, 2013a, p.5)	(Government of Ontario, 2018, §11:2-3)

While Ontario's new regulatory framework for homeopathy under the RHPA would certainly have been expected to comply with the Health Care Consent Act, such compliance would not have precluded CHO extending its informed consent policy requirements beyond those required under the Act (i.e., to include energetic risks). To do so would have brought the informed consent process into greater alignment with the concept of risk as understood by homeopaths themselves. That CHO's choice not to do so, despite HPRAC's prior advice, is epistemically-echoed more broadly across its policy documents, wherein biomedicine's scientific materialism ultimately dominates.

#### Homeopathic Compounding as CHO's Primary Material Risk

Given that Canada's federal Natural Health Products regulations already implement formal safeguards against toxicological harms from homeopathic products (Government of Canada, 2015), the CHO would have had no jurisdiction or mandate to intervene further in this regard. The potential “material” homeopathic harms relevant for CHO's work (and identified by HPRAC) would thus have been limited to: (a) allergy to the lactose or alcohol substrate of many homeopathic medicines; and (b) the potential for harms associated with homeopaths' individual compounding of remedies. While CHO nowhere explicitly addresses the issue of allergy as a form of material adverse events in policy, it does articulate a detailed practice standard on homeopathic “Compounding”. In this way, the CHO constitutes material risks of compounding as the only potential source of homeopathic adverse events in policy.

The CHO's compounding standard requires that homeopaths select raw medicinal materials with a “standard designation or equivalent pharmacopeia standard”; maintain “clean, sanitary, orderly” premises; employ “proper sanitary handling of materials” and implement various strategies to prevent “potential contamination” (College of Homeopaths of Ontario, 2018b, p. 3–4). This approach is to a large degree consistent with the WHO's guideline for the safe manufacture of homeopathic products. Notably, however, the CHO's compounding standard diverges from the WHO's (considerably lengthier) guideline by making no mention of quality control strategies pertaining to central energetic aspects of homeopathic compounding, such as the phases of dilution or succession required for remedy potentization. Indeed, the CHO's only references these and other concepts derived from homeopathic epistemology in its articulated competencies, far removed from tangible practice standards or guidelines that might give these concepts a palpable regulatory significance.

#### CHO's Policy Framework Minimizes Homeopathic Epistemology

Many of the CHO's required professional competencies for homeopaths are articulated in homeopathy-specific epistemic terms, using such terminology as the “law of similars” and “individualization of the case”; and, the CHO's safety competencies are no exception in this regard. One particular safety competency articulates an expectation that homeopath be proficient in responding to homeopathic aggravation, proving symptoms and disruption; the competency reads (with the present author's additions italicized in square parentheses):

Evaluate, interpret and adjust [the] treatment plan (e.g., second prescription) taking into consideration direction of cure, return of old symptoms [aggravation] and/or new symptomatology [proving symptoms, disruptions]. (p. 2.43).

As noted earlier, these homeopathic concepts have been described in the literature as potentially representing adverse effects (in the case of proving symptoms) or, if misinterpreted (in the cases of aggravation and disruption), as having the potential to precede harmful clinical omission.

That such matters are characterized as safety competencies suggests that the CHO intended to include homeopathy-specific risk conceptions in its risk management strategy for homeopaths. However, the CHO nowhere makes evident in its policy documents how it constitutes the risks associated with “return of old symptoms and/or new symptomatology.” Nor is it clear how the regulator expects homeopaths to manage such potential risks – except at their own discretion, as they would have done prior to statutory self-regulation. These policy gaps are remarkable given that two-thirds (*n* = 22) of CHO's safety competencies (*n* = 36) referred to knowledge- and skill-sets specific to the homeopathic profession. As the present analysis shows, the theoretical characterization of homeopathy-specific competencies as central to risk management is not tangibly reflected in the CHO's clinical standards and professional practice guidelines.

The aforementioned gaps are furthermore inconsistent with the degree of specificity that the CHO provides on other matters of professional conduct. A ten-page “interpretative [sic] guide,” for example, proposes to “assist[s] homeopaths understand the concept” of “professional [c]onflict of [i]nterest” (Transitional Council of the College of Homeopaths of Ontario, 2012b, p. 1). This document differentiates at length between “direct or indirect” types of conflicts, each with detailed subcategories (p. 1). Another practice standard elaborates instructions regarding professional “body language,” advising homeopaths to “[m]aintain appropriate and respective [sic] eye contact,” and noting that “physical gestures” should be used “with care [as] they may be interpreted differently than intended, based on each patient's unique culture” (College of Homeopaths of Ontario, 2013b, p. 2).

Setting aside the harms of unprofessional behavior, which are substantially addressed in the CHO's policy documents, the discussed gaps and inconsistencies ultimately affirm how unsubstantial Ontario's new regulatory framework for homeopathy regulations is, in terms of reducing the specific direct and indirect risks centralized in HPRAC's pre-regulatory recommendations. Like adverse events (which the CHO reduces to the issue of contaminated compounds), the CHO's handling of omission represents a significant simplification of HPRAC's analysis thereof.

#### Biomedically-Simplified Omission and Statutory Omission

Prevention of omission in the course of homeopathic care is variously addressed across the CHO's policy documents in ways that consistently draw attention to “material” epistemic considerations. “The most substantial policy document that provides specific direction in this regard is the CHO's Standard on Vaccination. This standard explicitly prohibits registrants from advising “patient[s] against vaccination” and using nosodes for homeoprophylactic purposes (College of Homeopaths of Ontario, 2015, p. 1). One safety competency is explicit that homeopaths should “recognize the signs and symptoms of potentially serious or life-threatening conditions to determine whether referral to other health-care professionals is needed.” However, no such standard specifies strategies for mitigating the homeopathy-specific forms of omission that may derive from unclear interpretation of particular energetic concepts – such as prolonged homeopathic aggravation or disruption. It is thus evident that despite the CHO's articulation of almost two-dozen safety competencies specific to the homeopathic profession, its tangible policy approaches emphasize those forms of material risk in which biomedical and homeopathic epistemology overlap. By doing so, the CHO's policies concurrently omit homeopathy-specific strategies for managing some of the risks identified as central factors driving the province's decision to regulate this particular T&CM occupational group.

## Discussion and Conclusion

Within a globalized context in which risk-based regulatory models have increasingly gained in importance (Lloyd-Bostock and Hutter, 2008; Phipps et al., 2011), the primary policy mandate underpinning the health professional regulatory statute in Ontario, Canada–the RHPA–is the reduction of potential patient harms. As such, state actors addressing the “problem” of regulating a new occupational group under the RHPA would necessarily construct their work in risk-focused discursive terms. Risk of course is no neutral concept. Further, the range of possible approaches to risk discourse construction in the context of T&CM professional regulation is especially complex given: (a) the biomedically-dominant context of dominant health systems; and (b) the differing (if at times overlapping) epistemic foundations of conventional biomedicine and many T&CM groups. The case of homeopathy's statutory regulation in Ontario, Canada shows how, even within a single regulatory process, distinct epistemic conceptions of risk may be differentially deployed by the state in response to various pressures or contextual factors, whether from stakeholders, political leadership or the content of statute itself. In this light, patient safety—constructed as policy's central driving factor—may be strategically volleyed about as a secondary priority.

Is homeopathy risky enough to regulate? This was the question posed of Ontario's Health Professions Regulatory Advisory Council (HPRAC), giving rise to a report advising that patient safety and the public interest would be indeed be served by the group's statutory self-regulation. However, the question of this group's statutory regulation carried epistemic pre-suppositions that would partially determine HPRAC's approach to framing its answer. As an occupational paradigm underpinned by vitalistic principles, and whose therapeutic agents are distinguished by their immateriality, homeopathy would be construed as scientifically implausible, and its practice as fundamentally unethical, by biomedical stakeholders. However, HPRAC's serious consideration of such an absolutist perspective from the outset would have fundamentally precluded any serious consideration of the political question posed by Ontario's Health Minister. To answer the Minister's question in good faith, and likely to align with his government's political agenda to proactively regulate T&CM professions, HPRAC would recognize but relegate the aforementioned views to its study's margins. Instead, HPRAC's 2006 report would explicitly centralize epistemic perspectives on risk considered more marginal within dominant biomedical health systems, but which aligned strongly with the perspectives of both users and providers of the T&CM therapy under study.

In the report, HPRAC initially frames its risk assessment for homeopathy using cultural safety principles, wherein which patients' cultural origins and practices, as well as clinical preferences, feature as primary considerations. Of particular note in this regard is homeopathy's widespread usage and long-standing statutory regulation across South Asia, from whence a significant number of Ontario's homeopathic practitioners and patients would have emigrated. As the report unfolds, Ontario homeopaths' voices—and their vitalistic perspectives–furthermore appear prominently, addressing both “material” concerns around remedy safety, professional conduct and omission, and homeopathic-specific epistemic concepts (e.g., proving symptoms, aggravations, disruption) that would be incongruous to biomedical critics. This same range of safety issues features prominently in HPRAC's final risk synthesis, used to substantiate its pro-regulatory position for the province's homeopaths. HPRAC thus suggests that in the interests of patient safety, the specific risks described from within homeopathy's vitalist epistemology warrant direct attention in policy.

However, Ontario's new regulatory framework for homeopathy would ultimately diverge from this recommendation, predominantly reflecting a set of “material” risk-related concerns. While the new homeopathy regulator would constitute a range of “safety competencies” that referred concurrently to homeopathic and biomedical epistemologies, it created no tangible policy mechanisms for addressing vitalistic safety considerations. Rather, policy frameworks focused on scientific materialist matters such as ingredient quality and hygiene in homeopathic compounding, and biomedical concerns such as the potential for deterred vaccination. Further entrenching scientific materialist epistemology into Ontario's regulatory framework for homeopaths, informed consent guidelines for the new professions explicitly constitute the potential for adverse outcomes in strictly “material” terms. While significantly at odds with HPRAC's prior recommendations, this approach is strongly aligned with the implicit epistemic underpinnings of Ontario's informed consent legislation. That being said, this policy is remarkable in that it precludes a requirement that patients be informed about the care they are receiving from the perspective of professionals who provide this care.

The present study, which analyses documentary data pertaining to Ontario's statutory regulation of homeopaths, is meant to provide a theoretical foundation for additional scholarship in the area of paradigm-specific risk conceptions. That being said, the case of homeopathy regulation in Ontario is in itself a distinctive policy exemplar, which would benefit from a more comprehensive analysis grounded in field data. The author's forthcoming publications will engage with more detailed analysis of survey and qualitative interview data pertaining to this particular practitioner group. Consistent with Bacchi's methodological approach to interpreting policy as discourse, it is important to note that this study's findings hold generalizable significance for the statutory regulation of various T&CM professionals worldwide. More specifically, the study speaks to the distinct epistemic challenges of governing T&CM practitioners in biomedically-dominant health systems contexts, as now discussed.

Setting aside the range of political motivations for and against such regulations, the work for instance indicates that biomedically-specific conceptions that constitute T&CM epistemologies as implausible and thus (axiomatically) as a source of unethical risk (“absolute omission”), must be to some degree marginalized in T&CM professional regulatory pursuits. This does not, of course, preclude a range of other “material” risk conceptions from being fruitfully addressed in policy. The current study also illuminates the ways in which T&CM professional regulatory processes may take place within a broader policy context underpinned by biomedicine's scientific materialist epistemology. At odds with the Ontario case however, and aligned with Bacchi's recommendation that policy alternatives be proposed in studies of this type, we contend that biomedically-dominant health systems contexts do not preclude vitalistic risk conceptions from being taken up in policy alongside more material considerations.

For example, Ontario's homeopathy regulator might have crafted a policy regarding homeopathic aggravation that followed Stub and colleagues' proposal to reduce associated risks by articulating clear parameters as to a reasonable associated severity and duration thereof (Stub et al., 2012a, 2015a). Why they did not do so, arguably at odds with the government's initial pro-regulatory justification for homeopathy, remains unclear in the analyzed documents themselves (but may become clearer in light of emerging field data being presently analyzed by the author and her research team). One possibility, seen in another case of T&CM professional regulation in Ontario (Ijaz and Boon, 2018a), is that the homeopathic regulatory team may have felt compelled by various political factors to align their policy framework closely with the biomedical parameters used by other professions in the province.

In addition, the regulator might have also required that informed consent documents for homeopathic patients include information about the potential for temporarily worsened symptoms after taking homeopathic remedies, reflecting homeopaths' own expectations of clinical outcomes and arguably improving patient safety. A related practice, which may admittedly “lead to a nocebo effect,” is common in Norwegian homeopathic practice, where “written information regarding homeopathic aggravation” is often provided “on the back of the prescription given to the patients” (Stub et al., 2015a). The absence of policies such as those aforementioned might from one vantage point be viewed as unethical, given that peer-reviewed scientific reporting suggests that aggravation-like episodes occur with some frequency in homeopathic clinical practice. However, there is another important argument for such policies as proposed above, which may prove compelling even to those who consider such vitalistic concepts as homeopathic aggravation to be bioscientifically implausible.

The key point here is that whether or not one views aggravation (for example) as plausible, that homeopaths themselves view it as such constitutes a source of risk (omission, in this case). In a sense the argument presented here represents a modified, or limited version of a biomedical discourse of “epistemology-as-risk.” By putting in place related regulatory controls (such as those proposed by Stub et al.), this risk is reduced in clearly defined ways by holding practitioners to particular standards of practice around a concept indigenous to their paradigm. Further, by formally informing patients about the defined parameters around which a particular vitalistic concept (e.g., aggravation) might be applied, they are likely to become more actively engaged participants in seeking out appropriate care as needed. Such a policy approach represents a regulatory application of the precautionary principle, which holds that where the risk of substantial harm exists, scientific uncertainty should not preclude pro-active measures being taken (de Sadeleer, 2006). This harm reduction principle has been widely applied in environmental and health law contexts across the globe, but to the author's knowledge has not yet been formally articulated in the context of T&CM professional regulation.

In contexts where differential concepts of omission are apparent between biomedical and T&CM epistemic paradigms, and in particular where T&CM epistemic conceptions provide additional nuance as to how the conditions of omission may specifically occur in clinical practice, it is imperative that such be explicitly incorporated into professional regulatory frameworks. As outlined above, the two central mechanisms for implementing such policy pertain involve explicit practice standards and guidelines that delineate the safe application of particular vitalistic concepts, paired with related patient consent stipulations.

With regards differential paradigm-specific conceptions of adverse events such as proving symptoms in homeopathy (Stub et al., 2015a); or symptom worsening after traditional Chinese acupuncture (Ijaz et al., 2016), appropriate policy responses would likely prove more complex. As in the case of omission, an ethics-based argument for informed consent may be predicated upon patients knowing how their health providers anticipate clinical care to (advantageously or detrimentally) proceed. As for the development of policy guidelines for investigating, substantially confirming and finally responding to such “energetically-explained” adverse events would present inevitable “material” challenges whose elaboration is beyond the current work's scope. However, the intellectual foundations of such policy may be found in an emerging body of T&CM-related clinical trials that track “energetic” adverse events alongside more “material” harms.

The decision whether to regulate a T&CM occupational policy is ultimately likely to be more political than strictly “evidentiary” in character, contingent on a complex range of contextual factors and forces. However, global health care contexts are increasingly characterized by cultural and epistemic pluralism; and T&CM practitioners remain vital in delivering both primary and complementary health care worldwide. If and when jurisdictional governments decide to move forward with T&CM professional regulations, epistemological purism and absolutism should be set aside in favor of pragmatism and inclusion aimed at enhancing practitioner delivery of, and patient consent to care that is both safe and informed. Until T&CM professional regulations accurately reflect both the material evidence for and vitalistic conceptualizations of potential harms associated with these occupations' concomitant treatments, it is unlikely that these goals will be fully realized.

## Data Availability Statement

All datasets analysed for this study are cited in the article.

## Author Contributions

The author confirms being the sole contributor of this work and has approved it for publication.

### Conflict of Interest

The author declares that the research was conducted in the absence of any commercial or financial relationships that could be construed as a potential conflict of interest.
